# Role of Chromatin and Epigenetic Dysregulation in Prostate Cancer: From Development to Progression and Therapeutic Response

**DOI:** 10.3390/cancers15235638

**Published:** 2023-11-29

**Authors:** Nikhila Paleati, Gnanasekar Munirathinam

**Affiliations:** 1 Department of Psychology and Neuroscience, College of Undergraduate Studies, Nova Southeastern University, Fort Lauderdale, FL 33328, USA; nikhilap2002@gmail.com; 2 Department of Biomedical Sciences, College of Medicine, University of Illinois, Rockford, IL 61107, USA

## 1. Introduction

A multitude of epigenetic modifications and genetic mutations have been found to play pivotal roles in the development and progression of prostate cancer (PCa). The review article of Kukkonen et al. [1] provides major insights into the mechanisms leading to prostate oncogenesis and the evolution of castration-resistant PCa (CRPC), with a particular emphasis on how the dysregulation of chromatin and epigenetic changes lead to carcinogenesis and tumor progression. Epigenetic variations can include non-coding RNA dysregulation, DNA methylation, and histone alterations. It is clearly necessary to understand these modifications because they can potentially serve as diagnostic markers for early detection of PCa and its prognosis, as well as risk assessment. The effect of epigenetic modification on PCa development can clearly be seen in the glutathione s-transferases 1 (GSTP1) gene, a key tumor suppressor gene. It was found that the hypermethylation of the promotor silences the GSTP1 gene. In addition to this, differences in gene expression patterns across different stages of PCa were associated with histone modifications and non-coding RNA dysregulation [1,2,3].

To further determine the importance of epigenetic modification in PCa malignancy, it is essential to view DNA methylation as a critical variable. As previously discussed, epigenetic suppression in PCa patients was observed following the promoter hypermethylation of GSTP1. Understanding this mutation allows clinicians to further analyze disease progression, using GSTP1 hypermethylation as a biomarker for PCa’s detection and for determining its suspected development path [3].

Along with DNA methylation, it was found that histone modifications serve as another method regulating PCa gene expression and chromatin structure. Specifically, variations in histone methylation and acetylation lead to epigenetic dysregulation, which contributes to differences in PCa malignancy across patients [4]. Non-coding RNAs and microRNAs further alter epigenetics in patients with PCa [5]. These components alter the biochemical pathways involved in PCa to alter tumor suppressor genes and further enhance carcinogenesis.

Changes in nucleosome positioning are another essential aspect of PCa evolution. Nucleosomes are largely known for their function as a fundamental structural unit of chromatin in eukaryotic cells. Along with their structural role, nucleosomes notably alter the genes involved in PCa. Nucleosome-remodeling complexes, such as switch/sucrose nonfermenting (SWI//SNF), shift the state of chromatin to enhance or inhibit gene expression. Through the SWI/SNF reading complex, chromatin accessibility is altered so as to shift the structure to a more relaxed state. The subsequent changes in gene expression allow for PCa to be classified as early-stage or late-stage CRPC [6,7].

The interaction between the aforementioned nucleosome-remodeling complex and androgen receptors (AR) is another crucial aspect of PCa malignancy. Interactions with androgen receptors activate key transcriptional factors that have been associated with the reprogramming of chromatin and progression to CRPC [8]. Previous research has highlighted the role of androgen receptors through evidence showing that androgen receptor signaling inhibitors (ARSIs) were effective in treating relapsed CRPC [9].

While new treatments are emerging, there are many aspects to consider when developing cancer therapies, especially the stage of the disease. The transcription of a myriad of critical genes is altered through differences in chromatin accessibility and remodeling complexes, which leads to the advancement of PCa. Variation in the levels of suppressed and accessible chromatin directly affects the balance of transcriptional factor activity. FOXA1 is one critical transcriptional regulator. FOXA1 functions to provide remodeling machinery and other advantageous components in order to suppress chromatin and, consequently, to encourage transcriptional factor binding. As more chromatin is altered to improve accessibility, the disease continues to progress to later, more severe stages [10,11].

From benign prostatic hyperplasia to development of CRPC, the patterns of chromatin accessibility are vastly different. The disparity in accessible chromatin across the different stages of PCa can be explained by the various enhancers in the expressed genes [11]. FOXA1, HOXB13, and androgen receptors are also markers that can be used to identify the stage of PCa. In terms of androgen receptors, ETS-related genes interact with these proteins and cause alterations in the functioning of transcription factors by blocking epithelial differentiation, reprogramming chromatin, and guiding enhancer activation [12].

The androgen receptor cistrome is also considerably altered during the development of PCa. The androgen receptor cistrome has been known to play an essential role in PCa’s initiation, as well as the evolution toCRPC. The changes in this structure have been associated with modified chromatin configuration, which leads to an enhanced availability of androgen receptor binding sites. The literature suggests that these modifications in structural states are a potential explanation for cells bypassing the effects of cancer treatment [10].

In addition to the importance of structural changes in nucleosome complexes and chromatin, the three-dimensional chromatin organization is another vital factor in PCa. In particular, topologically associated domains (TADs) and chromatin loops are noteworthy examples of 3D chromatin organization that affect gene regulation. Chromatin loops induce the organization of topologically associated domains while also bringing enhancers and promoters closer together so as to enable interaction in the chromatin space. TADs were also found to facilitate the constriction of chromatin loop movement [13].

Studies investigating structure have shown that 3D chromatin structure varies between normal and prostate-cancer-infected cells. For example, the copy number of topologically associated domains was significantly higher in patients diagnosed with PCa. This further drives the development of tumor growth and cancer stage progression, since the topologically associated domains have a multitude of regulatory features to drive cancer-related gene expression, such as increased numbers of androgen receptors, enhancers, and CCCTC-binding factors (CTCF) [14].

Likewise, chromatin loops can potentially regulate the presence of the androgen receptor cistrome. Protein–chromatin interactions, such as those between FOXA1, CTCF, and the androgen receptor, were investigated in a meta-analysis that emphasized the relationship between the expression of prostate-tissue-specific genes and chromatin loop structures. The essential function of loops in gene expression was further demonstrated through studies where the structure was disrupted, for example, through CTCF knockdown [15].

Detailed information about the effects of epigenetic and chromatin dysregulation provide researchers with the opportunity to gain a precise understanding of the differences between PCa stages. Evidence that can be used to classify the specific gene mutations and alterations has allowed clinicians to develop individualized treatment courses. One example can be found in the plausible therapeutic interventions discussed by researchers who investigated the role of SPOP mutations and the NSD gene family in PCa [11].

Epitranscriptomics, such as abnormal N6-methyladenosine regulation, are another essential aspect for understanding the influence of epigenetics on PCa progression [16]. Comprehension of the tumor microenvironment is another avenue which clinicians can pursue to develop treatments that can bypass resistance mechanisms. 

Current trials are ongoing to test medications known as epidrugs, highlighting the need for a more comprehensive understanding of PCa biomarkers. The use of RNA-based assays, such as BROMO-10, can help to classify molecular differences between disease progression stages [17]. By delving deeper into these distinctions, we could provide patients with more individualized, effective treatments for PCa.

## 2. Conclusions

The significant role of chromatin and epigenetic dysregulation in the progression of PCa from benign prostatic hyperplasia to CRPC is highlighted in the literature review of Kukkonen et al. [1]. Alterations in DNA methylation patterns, histone modifications, chromatin accessibility, nucleosome-remodeling complexes, chromatin 3D structure, and other aspects have all been associated with PCa malignancy. Identifying specific gene expression changes between primary PCa and CRPC is also crucial for developing an understanding of the mechanisms for treatment resistance. Overall, the authors of the article published herein [1] suggests that targeting these epigenetic and chromatin-affiliated modifications would allow for the creation of powerful treatment strategies or management methods that align with the patient’s unique PCa prognosis.
